# La discapacidad del jefe de familia aumenta la severidad de la inseguridad alimentaria en el hogar: análisis de una encuesta poblacional colombiana

**DOI:** 10.1590/0102-311XES208723

**Published:** 2024-08-19

**Authors:** Merari Fernandez-Perez, Juan Pablo Aparco, J. Jhonnel Alarco

**Affiliations:** 1 Disability Epidemiology Research Group, Universidad Científica del Sur, Lima, Perú.; 2 Centro Nacional de Alimentación Nutrición y Vida Saludable, Instituto Nacional de Salud, Lima, Perú.; 3 Facultad de Medicina, Universidad Nacional Mayor de San Marcos, Lima, Perú.

**Keywords:** Personas con Discapacidad, Seguridad Alimentaria, Estudios Transversales, Estudios Poblacionales en Salud Pública, Disabled Persons, Food Security, Cross-Sectional Studies, Population Studies in Public Health, Pessoas com Deficiência, Segurança Alimentar, Estudos Transversais, Estudos Populacionais em Saúde Pública

## Abstract

El objetivo de este estudio fue estimar la asociación entre la discapacidad del jefe de familia y la severidad de la inseguridad alimentaria de su hogar, en pobladores de Colombia, durante el 2017. Se realizó un análisis secundario de los datos de la *Encuesta Nacional de Calidad de Vida* del 2017 (ECV 2017) de Colombia. La variable independiente fue la discapacidad evaluada con las preguntas del grupo de Washington y la variable dependiente fue la inseguridad alimentaria medida con la *Escala Latinoamericana y Caribeña de Seguridad Alimentaria* (ELCSA). Se incluyeron variables de confusión sociodemográficas y relacionadas con la inseguridad alimentaria. Para demostrar la asociación se utilizó la regresión logística ordinal y se estimaron *odds ratio* (OR) con sus intervalos de 95% de confianza (IC95%). En todos los cálculos se consideró el muestreo complejo de la ECV 2017. Se incluyeron los datos de 8.488 jefes de familia. El 9,2% admitió que tenía alguna discapacidad y el 41,8% refirió que su hogar presentaba algún nivel de inseguridad alimentaria. Los hogares con un jefe de familia con discapacidad tuvieron 30% más probabilidad de mayor severidad de inseguridad alimentaria, en comparación con los hogares con un jefe de familia sin discapacidad (OR = 1,30; IC95%: 1,07-1,59), ajustado por múltiples variables de confusión. En conclusión, en Colombia, durante el 2017, la discapacidad de los jefes de familia aumentó la probabilidad de mayor severidad de la inseguridad alimentaria en sus hogares. Es necesaria la creación de programas de asistencia nutricional enfocados en las poblaciones vulnerables como las personas con discapacidad.

## Abstract (en)

This study investigated the association between head of household disability and the severity of food insecurity in Colombian households during 2017. A secondary data analysis was conducted based on 2017 *Colombian National Quality of Life Survey* (ECV 2017). As the independent variable, disability was assessed using questions from the Washington group, whereas the dependent variable - food insecurity - was measured by the *Latin American and Caribbean Food Security Scale* (ELCSA). Sociodemographic confounding variables and variables related to food insecurity were included. Association was analyzed by ordinal logistic regression, and the odds ratio (OR) was estimated with its 95% confidence intervals (95%CI). All calculations considered the complex sampling of ECV 2017. Data from 8,488 heads of household were included. A total of 9.2% of the participants had some type of disability and 41.8% reported some level of household food insecurity. Households with a head of household with some disability were 30% more likely to develop severe food insecurity compared with households with a head of household without disabilities (OR = 1.30; 95%CI: 1.07-1.59), adjusted for multiple confounding variables. In 2017, Colombian households with heads of household with disabilities were more likely to develop severe food insecurity. It is essential to implement nutritional assistance programs aimed at vulnerable populations, such as people with disabilities.

## Resumo

Este estudo busca determinar a associação entre a deficiência do chefe de família e a gravidade da insegurança alimentar em domicílios colombianos durante 2017. Foi realizada uma análise secundária dos dados da Pesquisa Nacional de Qualidade de Vida de 2017 (ECV 2017) da Colômbia. A variável independente foi a deficiência, avaliada por meio de perguntas do grupo de Washington, e a variável dependente foi a insegurança alimentar, medida pela *Escala de Segurança Alimentar da América Latina e do Caribe* (ELCSA). Foram incluídas variáveis de confusão sociodemográficas e relacionadas à insegurança alimentar. A regressão logística ordinal foi utilizada para analisar a associação, e a razão de probabilidades (OR) foi estimada com seus intervalos de 95% de confiança (IC95%). Em todos os cálculos foi considerada a amostragem complexa da ECV 2017. Foram incluídos dados de 8.488 chefes de família. Houve 9,2% dos participantes com algum tipo de deficiência e 41,8% relataram que seu domicílio apresentava algum nível de insegurança alimentar. Os domicílios com chefe de família com deficiência tiveram 30% mais chances de desenvolver insegurança alimentar mais grave, em comparação com as famílias com chefe de família sem deficiência (OR = 1,30; IC95%: 1,07-1,59), ajustado para múltiplas variáveis de confusão. Em 2017, os domicílios colombianos com chefes de família com deficiência tiveram maior probabilidade de desenvolver insegurança alimentar grave. É necessária a implementação de programas de assistência nutricional voltados para populações vulneráveis como as pessoas com deficiência.

## Introducción

Según datos actualizados de la Organización Mundial de la Salud (OMS) en el mundo viven 1.300 millones de personas con discapacidad, lo que representa el 16% del total de la población ^1^. Alrededor de 40 millones de personas (13,2% de la población) tienen discapacidad física, cognitiva o sensorial en Estados Unidos ^2^. En algunos países de América Latina, como Argentina, el 13% de las personas están en situación de discapacidad, de acuerdo con el Instituto Nacional de Estadística y Censos (INDEC) y su prevalencia es mayor en mujeres (14%). En el Perú, según el Instituto Nacional de Estadísticas e Informática (INEI), el 10,4% de la población tiene la condición de discapacidad ^3^. En Colombia, según los datos proporcionados por el Ministerio de Salud y Protección Social, existen 1.319.049 personas con discapacidad, lo que representa al 2,6% de toda la población ^4^.

La Organización de las Naciones Unidas para la Agricultura y la Alimentación (FAO) ^5^ define a la inseguridad alimentaria como la “*situación que existe cuando las personas carecen de acceso seguro a cantidades suficientes de alimentos inocuos y nutritivos para una vida activa y saludable*”. Según la OMS y la FAO, la inseguridad alimentaria moderada-grave afligió al 29,6% de la población mundial (2.400 millones de personas) en el 2022 ^6^. Según regiones, la inseguridad alimentaria tiene mayor prevalencia en África (60,9%), y América Latina y el Caribe (37,5%). Le siguen Asia (24,2%), América Septentrional y Europa (ambos con 8%) ^6^. Además, debido a la pandemia de la COVID-19, las cifras de inseguridad alimentaria se han incrementado en todo el mundo, con respecto al 2019 ^7^.

Las personas con discapacidad son más vulnerables a la inseguridad alimentaria por las desigualdades en educación y falta de empleo, lo que condiciona menores ingresos económicos ^8^, además tienen mayor gasto en salud por atenciones médicas frecuentes o por el uso de servicios de rehabilitación ^9^. Esta vulnerabilidad no se limita al acceso, sino a las restricciones para adquirir y preparar alimentos saludables, lo que aumenta el consumo de productos procesados y menos nutritivos ^10^. La mayoría de los estudios que han descripto este problema provienen de países desarrollados ^11^ ^,^ ^12^ ^,^ ^13^. En América del Sur y América Central la evidencia es limitada, la mayoría de los trabajos solo han determinado las prevalencias de inseguridad alimentaria, sin considerar su asociación con la discapacidad ^14^ ^,^ ^15^ ^,^ ^16^. Un estudio realizado en Brasil encontró una prevalencia moderada/grave de inseguridad alimentaria mayor a la población general ^9^.

Según nuestra revisión, existen pocos estudios con diferentes enfoques metodológicos y poco diferenciados que incluyen a las personas con discapacidad y la severidad de inseguridad alimentaria. La razón de incluir solo jefes de familia en este estudio radica en que son los principales proveedores económicos ^17^ y, debido a las barreras educativas, económicas, sanitarias y sociales que enfrentan las personas con discapacidad, la repercusión sería mayor sobre la seguridad alimentaria. Los hallazgos del presente análisis servirán para aumentar la evidencia sobre el real impacto de la inseguridad alimentaria en este grupo poblacional vulnerable, y para proponer medidas de protección alimentaria, priorizando aquellas familias que se encuentran en extrema pobreza y que dependen de una persona con discapacidad.

Por lo tanto, el objetivo primario de este estudio fue estimar la asociación entre la discapacidad de los jefes de familia y la inseguridad alimentaria de sus hogares, en pobladores de Colombia, durante el 2017. Nuestra hipótesis es que los hogares que tienen un jefe de familia con discapacidad tienen mayor probabilidad de inseguridad alimentaria. Los objetivos secundarios del estudio fueron evaluar la variabilidad de esta asociación, según sexo y grupos etarios.

## Métodos

### Diseño

Se llevó a cabo un análisis transversal de los datos secundarios de la *Encuesta Nacional de Calidad de Vida* del 2017 (ECV 2017) de Colombia. Se siguieron las pautas de la guía STROBE ^18^, para la redacción de este artículo.

### Contexto

La ECV 2017 fue realizada por el Departamento Administrativo Nacional de Estadística (DANE) de Colombia. Su objetivo fue “*obtener información para analizar y comparar las condiciones socioeconómicas de los hogares colombianos y aportar indicadores para la formulación y seguimiento de políticas públicas*” ^19^. Se aplicó durante siete semanas, desde el 1 de octubre al 15 de noviembre del 2017 y tiene representatividad nacional y regional ^19^. La ECV 2017 fue la última encuesta que incluyó la medición de la seguridad alimentaria, por esta razón se seleccionó este año para cumplir con el objetivo de la investigación.

El tamaño de la muestra de la ECV 2017 fue de aproximadamente 14.000 hogares. Para obtener representatividad nacional y regional, la ECV 2017 efectuó un muestreo complejo, que fue probabilístico, multietápico (vivienda, hogares y personas), estratificado (urbano y rural) y de conglomerados (10 viviendas contiguas).

Los encuestadores que participaron en la ECV 2017 fueron capacitados de forma virtual con herramientas multimedia sobre aspectos generales de la encuesta y sobre la recolección de los datos, y de forma presencial a través de talleres prácticos orientados a responder todas sus dudas. Además, fueron evaluados con dos pruebas de conocimientos. Previo a la aplicación del cuestionario, se realizó un reconocimiento y verificación de las manzanas seleccionadas con sus respectivas viviendas. También se hizo un proceso de sensibilización donde se visitó los hogares elegidos y se les informó que habían sido seleccionados para participar en la ECV 2017 ^19^.

La recolección de los datos se efectuó con el método de “barrido”, es decir, los equipos de campo aplicaron simultáneamente la encuesta hasta completar todos los hogares. La información fue recolectada directamente en un formulario electrónico, que luego se transfirió a la sede y subsedes del DANE. Este proceso se llevó a cabo diariamente a fin de garantizar el procesamiento continuo de los datos ^19^.

### Selección de participantes

Para el presente análisis se incluyeron solo a los jefes de familia, de 18 o más años, de ambos sexos, que participaron en la ECV 2017 y se excluyeron los individuos con datos faltantes o incongruentes.

### Variables

La variable dependiente fue la inseguridad alimentaria que se midió con la *Escala Latinoamericana y Caribeña de Seguridad Alimentaria* (ELCSA). Este instrumento es de ejecución rápida, tiene buena confiabilidad y adecuada validez ^20^. Se ha utilizado previamente en varios estudios en Colombia ^14^ ^,^ ^21^ ^,^ ^22^. Su uso es libre y no requiere ninguna licencia. Tiene 15 preguntas, que se dividen en dos secciones: la primera sección (preguntas 1 a 8) indaga sobre situaciones en el hogar que propician la inseguridad alimentaria en los adultos (mayores de 18 años) ocurrida en los últimos tres meses; y la segunda sección (preguntas 9 a 15) indaga sobre situaciones en el hogar que propician la inseguridad alimentaria en los menores de 18 años, ocurrida en los últimos tres meses. Las respuestas a las preguntas tienen cuatro alternativas: afirmativa (sí), negativa (no), no sabe (ns) y no responde (nr). Cada respuesta negativa vale 0 y cada respuesta afirmativa vale 1, los puntajes se suman y se clasifican en: hogar seguro (0 puntos), hogar con inseguridad leve (1 a 3 puntos), hogar con inseguridad moderada (4 a 6 puntos) y hogar con inseguridad severa (7 a 8 puntos). Para el presente estudio se consideró la primera sección (mayores de 18 años, por ser un criterio de inclusión). Finalmente, esta variable asumió cuatro categorías: sin inseguridad, inseguridad leve, inseguridad moderada e inseguridad severa.

La variable independiente fue la discapacidad, medida con las preguntas del grupo de Washington, utilizadas ampliamente para medir esta condición en encuestas poblacionales ^23^. La ECV 2017 empleó las siguientes preguntas: “Dada su condición física y mental, y sin ningún tipo de ayuda, puede: ¿Oír la voz o los sonidos? ¿Hablar o conversar? ¿Ver de cerca, de lejos o alrededor? ¿Mover el cuerpo, caminar o subir y bajar escaleras? ¿Agarrar o mover objetos con las manos? ¿Entender, aprender, recordar o tomar decisiones por sí mismo/a? ¿Comer, vestirse o bañarse por sí mismo(a)? ¿Relacionarse o interactuar con las demás personas? ¿Hacer las actividades diarias sin presentar problemas cardíacos, respiratorios?”, cuyas alternativas de respuestas son: “no puede hacerlo; sí, con mucha dificultad; sí, con alguna dificultad y sin dificultad”. Se asumió que el jefe de familia tenía la condición de discapacidad si respondió de forma afirmativa a alguna de las dos primeras alternativas, de las nueve preguntas descriptas anteriormente. Finalmente, esta variable asumió dos categorías: sin discapacidad y con discapacidad.

Como covariables se incluyeron al sexo (hombre y mujer), grupo etario (18-29, 30-44, 45-59 y 60 a más años), estado civil (casado/conviviente, viudo/separado/divorciado y soltero), nivel educativo (sin educación/preescolar, primaria, secundaria y superior), enfermedad crónica (no y sí), seguro de salud (no y sí), autopercepción de salud (muy bueno, bueno, regular y malo), consumo de tabaco (no y sí), autoidentificación étnica (ninguna, indígena y afrodescendiente), personas en el hogar (1 a 2, 3 a 4 y 5 a más), trabajo actual (no y sí), percepción de pobreza “¿Usted se considera pobre?” (no y sí), percepción de seguridad urbana “¿Cómo se siente en el barrio, pueblo o vereda donde vive?” (seguro e inseguro).

### Procesamiento y análisis estadístico

Los datos originales de la ECV 2017 se obtuvieron de la *web* de la DANE ^24^. Todos los cálculos se realizaron con el programa estadístico Stata SE versión 17 (https://www.stata.com). En el análisis descriptivo, las variables categóricas se mostraron como frecuencias y porcentajes. En el análisis bivariado, las diferencias según los niveles de inseguridad alimentaria se evaluaron con la prueba de chi cuadrado corregido con el estadístico F, por el diseño muestral. En el análisis multivariado, debido a que la inseguridad alimentaria tenía cuatro categorías ordenadas (sin inseguridad, inseguridad leve, inseguridad moderada e inseguridad severa) se decidió utilizar la regresión logística ordinal y reportar *odds ratio* (OR) con sus intervalos de 95% de confianza (IC95%). Las variables que resultaron asociadas significativamente (al menos en una categoría) en el modelo crudo se incluyeron en el modelo ajustado, de acuerdo con criterios estadísticos. Se uso un diagrama de bosque (*forest plot*) para visualizar los valores de OR de los modelos ajustados cuando se estratificó según sexo y grupos de edad (edad productiva de 18-59 años y adultos de 60 a más años) ^12^. Todos los cálculos se llevaron a cabo de acuerdo con el muestreo complejo de la ECV 2017. Un valor de p < 0,05 se consideró como significativo. La posible colinealidad entre las variables del modelo final se identificó con el cálculo manual del factor de inflación de la varianza (VIF) ^25^, de acuerdo con el diseño de encuestas. Se consideró un VIF ≥ 3, como indicio de multicolinealidad ^26^.

### Potencia estadística

Con la finalidad de determinar si el número de participantes seleccionados para el presente análisis era suficiente para estimar la asociación principal, se realizó un cálculo de potencia estadística. Del estudio de Brucker et al. ^27^ se obtuvo una prevalencia de 34,7% de inseguridad alimentaria y de la ECV 2017 se incluyeron 808 jefes de familia con discapacidad; del mismo estudio de Brucker et al. se obtuvo una prevalencia de 14,2% de inseguridad alimentaria y de la ECV 2017 se incluyeron 7.680 jefes de familia sin discapacidad ^27^. Este tamaño de muestra proporcionó una potencia estadística del 100% para demostrar la asociación entre la condición de discapacidad y la inseguridad alimentaria, de acuerdo con el programa OpenEpi 3.01 (http://www.OpenEpi.com).

### Aspectos éticos

El proyecto de investigación fue aprobado por la carrera de Medicina Humana de la Universidad Científica del Sur y fue exonerado de revisión por el comité de ética institucional por tratarse de datos de acceso público, según la resolución nº 012-DGIDI-CIENTÍFICA-2021. La base de datos de la ECV 2017 está disponible en la página *web* de la DANE y no es necesario ningún permiso para su descarga

## Resultados

**Figura 1:**
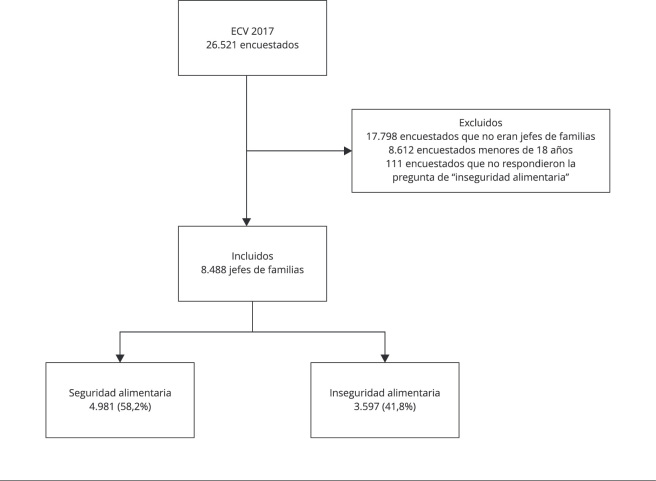
Flujograma de selección de participantes.

La base de datos contenía información de 26.521 personas. Se excluyeron a los que no eran jefes de familia, menores de 18 años y aquellos que no respondieron a la variable de interés (inseguridad alimentaria), quedando 8.488 jefes de familia para el análisis final (Figura 1).

ECV 2017: *Encuesta Nacional de Calidad de Vida* del 2017 de Colombia.

En el análisis descriptivo se encontró que el 9,2% de los jefes de familia refirió que tenía alguna discapacidad y el 41,8% indicó que su hogar tenía algún nivel de inseguridad alimentaria (24,7% inseguridad leve, 10,1% inseguridad moderada y 7% inseguridad severa). Asimismo, la mayoría de los jefes de familia eran hombres (57,3%), pertenecían al grupo etario de 30-44 años (32,1%), eran casados o convivientes (59,2%), tenían como máximo nivel educativo al secundario (39,4%), no padecían ninguna enfermedad crónica (79,5%), tenían seguro de salud (95%), percibían que su estado de salud era bueno (64%), vivían con 3 a 4 personas en su hogar (44%) y tenían un trabajo en el momento de la entrevista (64%). Asimismo, el 9,5% consumía tabaco, el 8,1% se identificaba como perteneciente a una etnia, el 24,3% percibía que era pobre y el 23,6% se sentía inseguro en la calle (Tabla 1).

**Tabla 1:** Características de los jefes de familia incluidos en el estudio y diferencias según discapacidad. Colombia, 2017 (n = 8.488).

Características	Total	Discapacidad del jefe de familia		Valor de p *
		No	Sí	
	n (%)	n (%)	n (%)	
Sexo	8.488 (100,0)	7.680 (90,8)	808 (9,2)	< 0,001
Hombre	4.793 (57,3)	4.424 (92,5)	369 (7,5)	
Mujer	3.695 (42,7)	3.256 (88,4)	439 (11,6)	
Grupo etario (años)				< 0,001
18-29	958 (12,9)	929 (97,0)	29 (3,0)	
30-44	2.511 (32,1)	2.406 (96,1)	105 (3,9)	
45-59	2.709 (30,1)	2.486 (91,4)	223 (8,6)	
60 a más	2.310 (24,9)	1.859 (79,9)	451 (20,1)	
Estado civil				< 0,001
Casado/Conviviente	4.911 (59,2)	4.541 (92,9)	370 (7,1)	
Viudo/Separado/Divorciado	2.328 (25,5)	1.974 (84,4)	354 (15,6)	
Soltero	1.249 (15,3)	1.165 (92,9)	84 (7,1)	
Nivel educativo				< 0,001
Sin educación/Preescolar	363 (3,7)	275 (76,4)	88 (23,6)	
Primaria	2.284 (23,7)	1.940 (84,1)	344 (15,9)	
Secundaria	3.352 (39,4)	3.121 (93,2)	231 (6,8)	
Superior	2.489 (33,2)	2.344 (94,3)	145 (5,7)	
Enfermedad crónica				< 0,001
No	6.673 (79,5)	6.267 (94,1)	406 (5,9)	
Sí	1.815 (20,5)	1.413 (77,8)	402 (22,2)	
Seguro de salud				
No	370 (5,0)	345 (93,0)	25 (7,0)	0,215
Sí	8.118 (95,0)	7.335 (90,6)	783 (9,4)	
Autopercepción de salud				< 0,001
Muy bueno	1.157 (15,8)	1.104 (95,5)	53 (4,5)	
Bueno	5.369 (64,0)	5.102 (95,1)	267 (4,9)	
Regular	1.822 (19,1)	1.404 (74,7)	418 (25,3)	
Malo	140 (1,1)	70 (50,0)	70 (50,0)	
Consumo de tabaco				0,788
No	7.718 (90,5)	6980 (90,8)	738 (9,2)	
Sí	770 (9,5)	700 (90,4)	70 (9,6)	
Autoidentificación étnica				0,809
Ninguna	7077 (91,9)	6.376 (90,8)	701 (9,2)	
Indígena	335 (1,6)	309 (89,1)	26 (10,9)	
Afrodescendiente	1076 (6,5)	995 (90,5)	81 (9,5)	
Personas en el hogar				< 0,001
1-2	3.391 (37,0)	3.008 (88,6)	383 (11,4)	
3-4	3.650 (44,0)	3.371 (92,9)	279 (7,1)	
5 o más	1.447 (19,0)	1.301 (90,2)	146 (9,8)	
Trabajo actual				< 0,001
No	3.045 (36,0)	2.519 (83,0)	526 (17,0)	
Sí	5.443 (64,0)	5.161 (95,1)	282 (4,9)	
Percepción de pobreza				< 0,001
No	6.019 (75,7)	5.524 (92,0)	495 (8,0)	
Sí	2.469 (24,3)	2.156 (96,9)	313 (13,1)	
Percepción de seguridad urbana				0,827
Seguro	6.753 (76,4)	6.114 (90,8)	639 (9,2)	
Inseguro	1.735 (23,6)	1.566 (90,6)	169 (9,4)	
Seguridad alimentaria				< 0,001
Segura	4.891 (58,2)	4.517 (92,5)	374 (7,5)	
Inseguridad leve	2.171 (24,7)	1.952 (90,5)	219 (9,5)	
Inseguridad moderada	837 (10,1)	724 (87,2)	113 (12,8)	
Inseguridad severa	589 (7,0)	487 (82,9)	102 (17,1)	

Nota: todos los porcentajes están ponderados según el diseño muestral de la *Encuesta Nacional de Calidad de Vida* del 2017 (ECV 2017) de Colombia.

* Prueba de chi cuadrado.

En el análisis bivariado, se halló que el 52,5% de los hogares con jefes de familia con discapacidad tenían algún nivel de inseguridad alimentaria en comparación con el 40,7% de los hogares con jefes de familia sin discapacidad. Asimismo, se encontró una mayor proporción de inseguridad alimentaria severa en los hogares de los jefes de familia con discapacidad (13%), en comparación con los hogares de los jefes sin discapacidad (6,4%). También se encontró una mayor proporción de inseguridad alimentaria moderada en los hogares de los jefes con discapacidad (14%) en comparación con los hogares de los jefes sin discapacidad (9,7%), siendo estas diferencias significativas (p < 0,001). Sin embargo, no se hallaron diferencias en las proporciones de inseguridad alimentaria leve entre los hogares de los jefes de familia con o sin discapacidad (25,5% y 24,6%, respectivamente) (Tabla 2).

**Tabla 2:** Diferencias según seguridad alimentaria en los jefes de familia de Colombia, 2017.

Características	Seguridad alimentaria				Valor de p *
	Seguro	Inseguridad leve	Inseguridad moderada	Inseguridad severa	
	n (%)	n (%)	n (%)	n (%)	
Discapacidad					< 0,001
No	4.517 (59,3)	1.952 (24,6)	724 (9,7)	487 (6,4)	
Sí	374 (47,5)	219 (25,5)	113 (14,0)	102 (13,0)	
Sexo					< 0,001
Hombre	2.932 (61,2)	1.176 (24,1)	418 (9,2)	267 (5,5)	
Mujer	1.959 (54,3)	995 (25,4)	419 (11,4)	322 (8,9)	
Grupo etario (años)					0,026
18-29	537 (56,1)	269 (28,6)	93 (8,6)	59 (6,7)	
30-44	1.383 (56,9)	684 (25,8)	259 (10,2)	185 (7,1)	
45-59	1.564 (57,4)	671 (23,8)	279 (11,3)	195 (7,5)	
60 a más	1.407 (62,1)	547 (9,3)	206 (9,3)	150 (6,3)	
Estado civil					< 0,001
Casado/Conviviente	2.875 (58,2)	1.303 (25,6)	480 (10,1)	296 (6,0)	
Viudo/Separado/Divorciado	1.242 (54,6)	625 (24,6)	254 (11,3)	213 (9,6)	
Soltero	802 (64,6)	271 (21,1)	113 (8,1)	84 (6,0)	
Nivel educativo					< 0,001
Sin educación/Preescolar	120 (33,0)	110 (27,7)	62 (19,7)	71 (19,2)	
Primaria	1.074 (45,7)	664 (27,8)	321 (16,0)	225 (10,6)	
Secundaria	1.871 (54,8)	911 (27,4)	340 (10,6)	230 (7,2)	
Superior	1.826 (74,2)	486 (18,8)	114 (4,3)	63 (2,7)	
Enfermedad crónica					0,014
No	2.292 (59,1)	1.247 (24,6)	1.604 (9,7)	1.530 (6,6)	
Sí	1.011(54,8)	328 (25,1)	239 (11,7)	237 (8,4)	
Seguro de salud					< 0,001
No	176 (47,3)	92 (23,5)	52 (12,9)	50 (16,3)	
Sí	4.715 (58,8)	2.079 (24,7)	785 (10,0)	539 (6,5)	
Autopercepción de salud					< 0,001
Muy bueno	820 (71,0)	222 (17,4)	71 (6,9)	44 (4,7)	
Bueno	3.245 (60,1)	1.349 (25,1)	486 (9,7)	289 (5,6)	
Regular	776 (42,7)	570 (29,7)	253 (15,4)	223 (12,2)	
Malo	50 (38,0)	30 (17,6)	27 (18,3)	33 (26,0)	
Consumo de tabaco					< 0,001
No	4.492 (59,0)	1.966 (24,5)	754 (9,9)	506 (6,6)	
Sí	399 (50,8)	205 (26,6)	83 (12,4)	83 (10,2)	
Autoidentificación étnica					< 0,001
Ninguna	4.173 (59,7)	1.853 (24,7)	655 (9,5)	396 (6,1)	
Indígena	193 (37,5)	80 (32,5)	34 (17,2)	28 (12,8)	
Afrodescendiente	525 (43,7)	238 (22,5)	148 (16,6)	165 (17,2)	
Personas en el hogar					< 0,001
1-2	2.220 (67,0)	728 (20,4)	255 (7,0)	188 (5,5)	
3-4	2.032 (56,5)	1.038 (27,1)	357 (10,3)	223 (6,0)	
5 o más	639 (45,4)	405 (27,3)	225 (15,5)	178 (11,9)	
Trabajo actual					< 0,001
No	1.632 (54,5)	779 (24,7)	359 (11,8)	275 (9,0)	
Sí	3.259 (60,3)	1.392 (24,7)	478 (9,1)	314 (5,8)	
Percepción de pobreza					< 0,001
No	4.031 (66,6)	1.413 (22,8)	385 (7,0)	190 (3,6)	
Sí	860 (32,3)	758 (30,4)	452 (19,8)	399 (17,5)	
Percepción de seguridad urbana					< 0,001
Seguro	4.044 (60,5)	1.720 (24,7)	590 (8,9)	399 (5,9)	
Inseguro	847 (51,2)	451 (24,5)	247 (14,0)	190 (10,3)	

Nota: todos los porcentajes están ponderados según el diseño muestral de la *Encuesta Nacional de Calidad de Vida* del 2017 (ECV 2017) de Colombia.

* Prueba de chi cuadrado.

En el análisis multivariado, en el modelo crudo, los hogares que tienen un jefe de familia con discapacidad presentaron 73% más probabilidad de mayor inseguridad alimentaria en comparación con los hogares cuyos jefes no tienen discapacidad (OR = 1,73; IC95%: 1,44-2,07). En el modelo ajustado por sexo, grupo etario, estado civil, nivel educativo, enfermedad crónica, seguro de salud, autopercepción de salud, consumo de tabaco, autoidentificación étnica, personas en el hogar, percepción de pobreza y percepción de seguridad urbana, los hogares que tienen un jefe de familia con discapacidad presentaron 30% más probabilidad de mayor inseguridad alimentaria en comparación con los hogares cuyos jefes no tienen discapacidad (OR = 1,30; IC95%: 1,07-1,59) (Tablas 3 y 4).

**Tabla 3:** Análisis multivariado entre la discapacidad del jefe de familia y la inseguridad alimentaria de su hogar. Colombia, 2017.

Características	Modelo crudo	Valor de p	Modelo ajustado *	Valor de p
	OR (IC95%)		OR (IC95%)	
Discapacidad				
No	Referencia		Referencia	
Sí	1,73 (1,44-2,07)	< 0,001	1,30 (1,07-1,59)	0,008

IC95%: intervalo de 95% de confianza; OR: *odds ratio*.

* Modelo ajustado por sexo, grupo etario, estado civil, nivel educativo, enfermedad crónica, seguro de salud, autopercepción de salud, consumo de tabaco, autoidentificación étnica, personas en el hogar, percepción de pobreza y percepción de seguridad urbana.

**Tabla 4:** Análisis multivariado que incluye el modelo crudo y ajustado de todas las variables incluidas en el estudio. Colombia, 2017.

Características	Modelo crudo	Valor de p	Modelo ajustado	Valor de p
	OR (IC95%)		OR (IC95%)	
Discapacidad				
No	Referencia		Referencia	
Sí	1,73 (1,44-2,07)	< 0,001	1,30 (1,07-1,59)	0,008
Sexo				
Hombre	Referencia		Referencia	
Mujer	1,37 (1,23-1,52)	< 0,001	1,20 (1,05-1,38)	0,010
Grupo etario (años)				
18-29	Referencia		Referencia	
30-44	1,01 (0,84-1,20)	0,942	0,84 (1,05-1,38)	0,085
45-59	1,01 (0,84-1,21)	0,899	0,68 (0,55-0,84)	< 0,001
60 a más	0,82 (0,69-0,99)	0,039	0,40 (0,31-0,50)	< 0,001
Estado civil				
Casado/Conviviente	Referencia		Referencia	
Viudo/Separado/Divorciado	1,22 (1,08-1,38)	0,002	1,23 (1,05-1,45)	0,011
Soltero	0,78 (0,67-0,92)	0,004	1,07 (0,87-1,30)	0,533
Nivel educativo				
Sin educación/preescolar	Referencia		Referencia	
Primaria	0,55 (0,41-0,72)	< 0,001	0,66 (0,50-0,88)	0,004
Secundaria	0,36 (0,27-0,48)	< 0,001	0,45 (0,34-0,60)	< 0,001
Superior	0,15 (0,11-0,20)	< 0,001	0,25 (0,19-0,34)	< 0,001
Enfermedad crónica				
No	Referencia		Referencia	
Sí	1,22 (1,07-1,39)	0,003	0,99 (0,84-1,16)	0,887
Seguro de salud				
No	1,82 (1,38-2,40)	< 0,001	1,48 (1,09-2,00)	0,011
Sí	Referencia		Referencia	
Autopercepción de salud				
Muy bueno	Referencia		Referencia	
Bueno	1,57 (1,31-1,87)	< 0,001	1,24 (1,03-1,50)	0,026
Regular	3,25 (2,66-3,98)	< 0,001	1,93 (1,54-2,43)	< 0,001
Malo	5,70 (3,34-9,73)	< 0,001	2,75 (1,62-4,65)	< 0,001
Consumo de tabaco				
No	Referencia		Referencia	
Sí	1,43 (1,19-1-71)	< 0,001	1,30 (1,07-1,56)	0,007
Autoidentificación étnica				
Ninguna	Referencia		Referencia	
Indígena	2,37 (1,74-3,22)	< 0,001	1,60 (1,13-2,25)	0,008
Afrodescendiente	2,27 (1,83-2,83)	< 0,001	1,65 (1,32-2,05)	< 0,001
Personas en el hogar				
1-2	Referencia		Referencia	
3-4	1,51 (1,34-1,70)	< 0,001	1,51 (1,32-1,74)	< 0,001
5 o más	2,53 (2,16-2,95)	< 0,001	2,0 (1,68-2,38)	< 0,001
Trabajo actual				
No	Referencia		Referencia	
Sí	0,76 (0,68-0,85)	< 0,001	0,87 (0,75-1,01)	0,070
Percepción de pobreza				
No	Referencia		Referencia	
Sí	4,53 (4,01-5,12)	< 0,001	3,11 (2,73-3,55)	< 0,001
Percepción de seguridad urbana				
Seguro	Referencia		Referencia	
Inseguro	1,56 (1,38-1,77)	< 0,001	1,47 (1,29-1,68)	< 0,001

IC95%: intervalo de 95% de confianza; OR: *odds ratio*.

**Figura 2:**
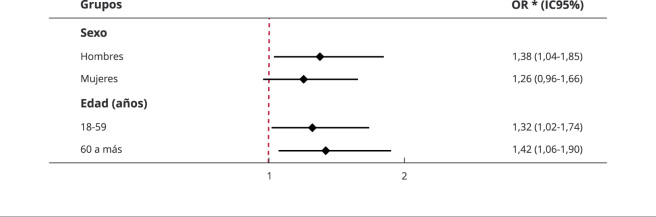
Estratificación del modelo ajustado según sexo y grupo etario.

Cuando se estratificó según grupo etario, se encontró que la asociación entre la condición de discapacidad y la inseguridad alimentaria estuvo presente en los jefes de familia en edad productiva (19-59 años) (OR = 1,32; IC95%: 1,02-1,74), y en los adultos mayores (60 a más años) (OR = 1,42; IC95%: 1,07-1,90). Cuando se estratificó según sexo, se encontró que la asociación solo estuvo presente en los jefes de familia hombres (OR = 1,38; IC95%: 1,04-1,85), al parecer, en las mujeres, la discapacidad no condiciona a un mayor riesgo de inseguridad alimentaria (Figura 2).

IC95%: intervalo de 95% de confianza; OR: *odds ratio*. * Modelo ajustado por sexo, grupo etario, estado civil, nivel educativo, enfermedad crónica, seguro de salud, autopercepción de salud, consumo de tabaco, autoidentificación étnica, personas en el hogar, percepción de pobreza y percepción de seguridad urbana.

No se halló indicios de multicolinealidad entre las variables del modelo ajustado (VIF ≈ 2).

## Discusión

En el presente estudio se encontró que el 9,2% de los jefes de familia refirió que tenía alguna discapacidad y el 41,8% de los hogares estaban en riesgo de inseguridad alimentaria. Asimismo, el 52,5% de los hogares con jefes de familia con discapacidad tenían algún nivel de inseguridad alimentaria. Luego de ajustar por múltiples variables de confusión, las probabilidades de mayor severidad de inseguridad alimentaria aumentaron en los hogares que tenían un jefe de familia con discapacidad. Esta asociación se mantuvo en los hogares cuyos jefes eran hombres, se encontraban en edad productiva o tenían de 60 a más años.

Se halló una prevalencia del 41,8% de inseguridad alimentaria. Otros trabajos realizados en Colombia han descripto valores similares. Por ejemplo, un estudio que usó el mismo instrumento y que analizó los datos de la *Encuesta Nacional de Situación Nutricional* (ENSIN) de 2015 encontró una prevalencia del 54,2% de inseguridad alimentaria ^28^. Un trabajo que analizó los datos del Gallup World Poll en una muestra representativa de 1.975 mayores de 15 años de Colombia, encontró una prevalencia de inseguridad alimentaria del 40% en el 2019 ^29^. Estas prevalencias de inseguridad alimentaria son tan altas como las descriptas en migrantes y refugiados del Oriente Medio y África, que oscilan entre 40% y 71%, respectivamente ^30^ o del 60,9% en los hogares urbanos de África Oriental ^31^. Posiblemente, en la actualidad estas cifras se han incrementado, debido a las restricciones durante la pandemia de la COVID-19, donde disminuyó el acceso a los alimentos ^7^. Al respecto, un estudio realizado en más de un millón de personas de todo el mundo reportó una frecuencia de 76,8% de inseguridad alimentaria para Colombia, durante la pandemia de la COVID-19 ^32^.

El 52,5% de los hogares con jefes de familia con discapacidad tenían algún nivel de inseguridad alimentaria. Un estudio poblacional brasileño realizado en 1.251 hogares que recibían el Beneficio de Pago Continuo, un beneficio económico que consiste en un salario mínimo para personas con discapacidad encontró que el 40,8% de los hogares con personas con discapacidad estaban en situación de inseguridad alimentaria ^9^. Este trabajo es uno de los pocos en la región que evalúa la proporción de inseguridad alimentaria en hogares con personas con discapacidad. Otro estudio que incluyó una muestra representativa de 205 personas con discapacidad de 14-64 años de la ciudad de Durame, al sureste de Etiopía, encontró una proporción de 96% de inseguridad alimentaria ^33^. Como se puede apreciar, las proporciones de inseguridad alimentaria en las personas con discapacidad varían de acuerdo con el país en donde se lleva a cabo el estudio, posiblemente influenciado por el entorno socioeconómico, entre una de las principales causas.

El hallazgo principal de este estudio muestra que los hogares con un jefe de familia con discapacidad tienen mayor probabilidad de inseguridad alimentaria más grave. Al respecto, la evidencia apoya estos resultados. Por ejemplo, en una revisión de alcance que incluyó artículos publicados entre 1966 y 2018 (50 años), de cinco importantes bases de datos, fueron encontrados 32 trabajos que evaluaron estadísticamente esta asociación. Dicha investigación demostró que la discapacidad se asocia con un mayor riesgo de inseguridad alimentaria en el hogar, en diferentes poblaciones y entornos geográficos. Es necesario mencionar que esta revisión solo incluyó artículos en inglés y ninguno de Latinoamérica ^10^.

Otros trabajos publicados luego de esta revisión también han descripto resultados similares. Por ejemplo, un estudio que utilizó los datos de 2.654 hogares de Corea del Sur que participaron en la *Encuesta Nacional de Examen de Salud y Nutrición de Corea* (KNHANES) del 2013 encontró que la condición de discapacidad de algún miembro del hogar está estrechamente relacionada con el estado de seguridad alimentaria. Los hogares con miembros con discapacidad mental, discapacidad de órganos internos (disfunción renal, cardíaca, respiratoria o hepática; fístula intestinal/urinaria; o epilepsia) o discapacidad grave, tuvieron mayores probabilidades de inseguridad alimentaria ^11^. Otro estudio que evaluó la asociación entre ciertos tipos de discapacidad y la inseguridad alimentaria en adultos en edad productiva y en adultos mayores de Estados Unidos usando datos del *Encuesta Nacional de Examen de Salud y Nutrición* (NHANES) de 1999 a 2014 encontró que las limitaciones para trabajar, limitaciones funcionales y limitaciones para administrar el dinero se asocian con una mayor probabilidad de inseguridad alimentaria. Además, las limitaciones para moverse, ver y oír aumentan la inseguridad alimentaria, pero solo en los adultos en edad productiva ^12^.

Por su parte, otra investigación que evaluó la asociación entre el número de discapacidades y la inseguridad alimentaria, con los datos de la quinta ola de la encuesta *Alimentos y Usted* de la Agencia de Normas Alimentarias del Reino Unido del 2018, encontró que, tanto el número y el tipo de discapacidad predijeron la inseguridad alimentaria. Este trabajo halló que cada discapacidad adicional se asoció con mayores probabilidades de inseguridad alimentaria (OR = 1,60; IC95%: 1,40-1,83) y que las personas con discapacidades físicas y mentales tenían mayores probabilidades de inseguridad alimentaria grave (OR = 8,97; IC95%: 3,54-22,7) ^13^. Además, un estudio transversal realizado en Etiopía, entre diciembre del 2014 y agosto del 2015, cuyo objetivo fue comparar los niveles de inseguridad alimentaria en las personas con discapacidad mental en un entorno rural africano con alta carga de inseguridad alimentaria; encontró que el 32,5% de las personas con discapacidad informaron inseguridad alimentaria en sus hogares en comparación con el 15,9% reportado por las personas sin discapacidad (OR = 2,82) ^34^.

La alta prevalencia de inseguridad alimentaria hallada en el presente estudio podría deberse al conflicto armado que ha durado más de 50 años en Colombia y que ha provocado la muerte de 220.000 personas y el desplazamiento del 10% de la población (aproximadamente seis millones de personas), siendo uno de los grupos de desplazados más grandes del mundo ^29^. Pese a que en el 2016 se firmó el acuerdo de paz entre el gobierno y las Fuerzas Armadas Revolucionarias de Colombia (FARC), las prevalencias de inseguridad alimentaria se han incrementado en los últimos años, pasando de un 33% en el 2016 a un 40% en el 2019 ^29^. Esto ha ocasionado una inseguridad alimentaria “sostenida”, con mayor prevalencia en las zonas rurales y en los hogares encabezados por mujeres. Sin embargo, el autoconsumo parece mitigar el efecto perjudicial de la inseguridad alimentaria en las zonas rurales; y las mujeres tendrían mayor éxito en proteger la seguridad alimentaria de sus hogares ^28^. Además, debido a las transiciones nutricionales en Colombia, se ha observado que la inseguridad alimentaria está relacionada con un menor consumo de alimentos saludables y con un mayor consumo de alimentos ricos en energía, lo que ha ocasionado retraso en el crecimiento de los niños, y sobrepeso y obesidad en sus madres, lo que se conoce como la “doble carga de malnutrición” ^21^.

Los resultados del presente estudio y de la evidencia revisada muestran que las familias con un jefe con discapacidad tienen más probabilidades de padecer mayor severidad de inseguridad alimentaria. La discapacidad está asociada fuertemente con las condiciones socioeconómicas de las personas ^35^ ^,^ ^36^, las que influyen directamente en el acceso a los alimentos de calidad ^37^. Es decir, las personas con discapacidad son, en general, más pobres y, por lo tanto, sus familias estarían en mayor riesgo de inseguridad alimentaria ^10^.

Los hallazgos secundarios de esta investigación indican que esta asociación resultó significativa en los jefes de familia en edad productiva y en los de 60 a más años, aunque en este último grupo, la magnitud de la asociación fue mayor. Se conoce que la discapacidad se incrementa con la edad, ya que existe una mayor prevalencia de discapacidad en las personas de 65 a más años ^38^. Esta asociación directa entre la edad y la discapacidad es tan fuerte que incluso cambia la proporción de personas de 60 a más años frente a la inseguridad alimentaria, hallado en este estudio. De igual forma, la mayor probabilidad de inseguridad alimentaria solo estuvo presente cuando los jefes de familia con discapacidad eran hombres. Al parecer, las limitaciones para acceder a un empleo con una remuneración adecuada, podría explicar este resultado. Otras razones que expliquen una mayor inseguridad alimentaria en las familias de los jefes varones podrían estar relacionadas con las enfermedades crónicas, los vicios, pobre educación, pobreza, entre otros factores descriptos secundariamente en este trabajo.

Los resultados de este estudio están en concordancia con la evidencia disponible; como ya se mencionó, la mayoría de los trabajos que han abordado esta asociación fueron realizados en países desarrollados, por esta razón, estos hallazgos son relevantes para la implementación de políticas públicas de alimentación en Colombia. Varios países de Latinoamérica ofrecen subvenciones económicas a las personas con discapacidad, como Chile, donde funciona el Programa de Pensiones Solidarias que atiende esta población, o en Brasil, donde existe el programa Beneficio de Pago Continuo que brinda una transferencia mensual de dinero a las personas con discapacidad pobres o de 60 a más años ^39^. Asimismo, existen programas de asistencia nutricional para los familiares más vulnerables (especialmente los niños), que dependen de un jefe de familia con discapacidad, como el Programa de Asistencia Nutricional Suplementaria (SNAP) que proporciona asistencia alimentaria a 11 millones de personas con limitaciones funcionales, laborales o que ya reciben beneficios por el gobierno federal de Estados Unidos ^40^. Otro ejemplo de la implementación de un programa de ayuda alimentaria que podría beneficiar a niños en hogares cuyo jefe tiene discapacidad es Qali Warma que consiste en la entrega de desayunos a los niños de inicial y primaria de las zonas más pobres del Perú, con la intención de suplir los requerimientos energéticos para lograr un desarrollo físico y mental adecuado ^41^; aunque este programa no es específico para niños con padres con discapacidad, estos son incluidos. La implementación de programas similares enfocados en los hogares dependientes de adultos con discapacidad podría contrarrestar el efecto perjudicial de la falta de alimentos o de la mala alimentación, demostrada en este estudio y en los demás trabajos revisados.

### Limitaciones y fortalezas

Se deben reconocer las siguientes limitaciones: la evaluación de la discapacidad por preguntas autorreferidas, estaría influenciada por el sesgo de deseabilidad social, lo que podría incrementar la prevalencia de la discapacidad. Algunas variables relacionadas con la inseguridad alimentaria, como los ingresos económicos ^42^, los beneficios sociales ^9^, la ansiedad o depresión ^43^ y el consumo de alcohol ^44^, no estaban disponibles en la base analizada. No se evaluó el posible efecto de los tipos de discapacidad sobre la inseguridad alimentaria, debido a las pocas observaciones que tenían estas variables. Por el diseño transversal del estudio no se puede afirmar la existencia de “causalidad” entre las variables principales. Como fortaleza se debe mencionar que este estudio es pionero en analizar esta asociación en población colombiana; además, la ECV 2017 es una encuesta poblacional, por lo que los resultados del presente análisis son representativos de la población estudiada.

## Conclusiones

En Colombia, durante el 2017, los hogares con un jefe con discapacidad tuvieron más probabilidades de tener mayor severidad de inseguridad alimentaria, en comparación con las familias cuyo jefe no tenía discapacidad. Además, la discapacidad aumentó las probabilidades de inseguridad alimentaria más grave en los jefes de familia varones, en edad productiva y en adultos de 60 a más años. Estos resultados muestran un problema muy poco visualizado en los países en desarrollo como Colombia. Es necesario que los programas nutricionales protejan a las poblaciones vulnerables, como las personas con discapacidad y sus familias frente a la inseguridad alimentaria.
